# How can a remotely delivered, personalised physical activity intervention for people with high risk of breast, lung and bowel cancer recurrence be implemented in England? Protocol for a mixed-methods process evaluation embedded in a feasibility basket trial

**DOI:** 10.1136/bmjopen-2025-112103

**Published:** 2026-05-24

**Authors:** Gillian Jackson, Mark Pearson, Alex F Bullock, Judith Cohen, Chao Huang, Michael Lind, John Saxton, Caroline Wilson, Maureen Twiddy, Cynthia C Forbes

**Affiliations:** 1 Hull York Medical School, Kingston Upon Hull, University of Hull, Hull, England, UK; 2 School of Sport, Exercise, and Rehabilitation Sciences, University of Hull, Hull, England, UK; 3 The Christie NHS Foundation Trust, Manchester, England, UK

**Keywords:** PUBLIC HEALTH, Implementation Science, Protocols & guidelines, Cancer Survivors, Exercise

## Abstract

**Introduction:**

Randomised controlled trials (RCTs) are essential to determine intervention effectiveness yet they often fail to capture how and why interventions succeed or fail in different contexts. Embedding a process evaluation alongside a clinical trial allows exploration of implementation processes, intervention fidelity and contextual influences. The CANFit trial is a basket-design RCT evaluating a personalised, remotely delivered exercise intervention for people diagnosed with breast, lung and bowel cancer with increased risk of recurrence. This embedded process evaluation aims to understand how individual, team and organisational factors influence intervention delivery and uptake.

**Methods and analysis:**

A concurrent, mixed-methods process evaluation will be conducted using a hybrid type 1 design. Data will be collected from multiple sources, including participant and trainer questionnaires, semi-structured interviews, intervention adherence logs, trainer diaries and observations. Five core implementation outcomes, guided by Proctor’s framework—acceptability, appropriateness, fidelity, penetration and sustainability—will structure the evaluation. Quantitative data will be analysed descriptively and qualitative data will undergo framework analysis using both deductive and inductive coding. Data integration will occur through a convergent mixed-methods approach, using context-mechanism-outcome (CMO) configurations to refine programme theory.

**Ethics and dissemination:**

Ethical approvals were obtained through Hull York Medical School (ID: 23/SS/0060) and the UK NHS Health Research Authority (ID: 327663). All participants will provide informed consent before taking part. Data will be handled according to General Data Protection Regulation and University of Hull data management policies. Findings will be disseminated through peer-reviewed publications, conference presentations, stakeholder reports and lay summaries for participants and the public.

**Trial registration number:**

ISRCTN97662203.

**STRENGTHS AND LIMITATIONS OF THIS STUDY**

- The process evaluation is guided by established frameworks (Proctor’s implementation outcomes and Lengnick-Hall’s taxonomy, ensuring conceptual clarity and standardised reporting.
- The hybrid type 1 design supports concurrent evaluation of implementation and intervention delivery.
- The process evaluation team is conducted by an independent team, ensuring objectivity while maintaining contextual insight.
- The study is limited to participating oncology services, which may reduce wider applicability.
- The qualitative sample is relatively small and restricted to individuals approached for the feasibility trial, which may constrain exploration of some aspects of diversity.

## Introduction

 Randomised controlled trials (RCTs) are often described as the gold standard for evaluating intervention effectiveness.^1^,^3^ However, although RCTs are critical in determining efficacy, they can be limited in explaining how and why interventions succeed or fail, especially in complex healthcare settings. Understanding both the outcomes and the processes underlying them is crucial, particularly when interventions are implemented across diverse healthcare environments. Process evaluations within RCTs provide a mechanism to explore these issues, offering insights into implementation fidelity, acceptability, dosage and contextual factors that influence outcomes.^4^ These evaluations can support interpretation of trial results and offer critical information about why an intervention was or was not effective.^5^ The importance of integrating a process evaluation into clinical trials is now widely recognised.^6^

A significant challenge in implementation research is distinguishing between the success of an intervention’s clinical outcomes and the success of its delivery.^1^ Favourable trial outcomes can mask underlying implementation failures, or, conversely, poor outcomes may result from failed delivery rather than the intervention itself. Therefore, it is essential to separately evaluate implementation processes and outcomes. Proctor *et al* proposed a framework identifying eight core implementation outcomes: acceptability, adoption, appropriateness, feasibility, fidelity, cost, penetration and sustainability that help in assessing implementation independently of clinical efficacy.^1^ In this study, we focus on five of these outcomes: acceptability, appropriateness, fidelity, penetration and sustainability as they are most relevant to the trial context and feasibility stage.

Guided by realist principles,^7^ this process evaluation aims to explore how and why the intervention produces (or does not produce) its intended outcomes in different contexts, across three cancer types: breast, bowel and lung. Realist evaluation emphasises the interaction between context, mechanisms and outcomes, helping to identify for whom an intervention works, under what circumstances, and why.^7^

We present here the protocol for a mixed-methods process evaluation embedded within the CANFit (trial name, not an acronym) randomised controlled trial (RCT) feasibility, designed to evaluate both the effectiveness and the implementation of a remotely delivered, personalised exercise programme for people diagnosed with cancer.

### The CANFit randomised feasibility trial

Yorkshire has some of the highest cancer diagnosis and mortality rates in England,^8^ with lung, breast and bowel cancers being particularly prevalent.^9^ Despite improvements in treatment, there is an urgent need to focus on enhancing post-treatment recovery and quality of life for people diagnosed with cancer.^10^ Physical activity has demonstrated promise in reducing side effects and improving long-term outcomes among cancer patients.^11^ Although much of the evidence to date comes from observational studies, findings suggest that regular moderate-to-vigorous physical activity (defined as ≥150 min of moderate-intensity activity per week)^12^ is associated with improved survival in certain cancers, particularly breast and bowel cancers.^13 14^

The CANFit randomised feasibility trial^15^ will assess a personalised, multicomponent, remotely delivered physical activity intervention supported by trained exercise professionals. Participants will be compared against controls receiving either general advice (Macmillan Cancer Support resources) or standard care at their respective NHS Trusts. The trial targets post-treatment people diagnosed with cancer at increased risk of recurrence and is designed to inform the development of a definitive, large-scale RCT.

A novel basket trial design is used, applying a single protocol across three distinct tumour groups.^16^ This allows for efficient data collection and cross-cancer comparisons, enhancing the trial’s robustness. The study will evaluate whether structured, personalised physical activity can contribute to healthier, longer lives free from recurrence.

### The CANFit intervention

As detailed in the main protocol,^15^ the intervention offers tailored physical activity prescriptions based on participants’ baseline assessments, specific needs and participants’ available resources. It includes behaviour change techniques such as goal setting, barrier identification and self-monitoring, delivered via remote counselling by trained exercise professionals. Support is initially intensive, with three contacts per week, before tapering over 6 months. Participant engagement is supported through a range of materials, including digital and paper-based formats depending on context and accessibility.

Participants are encouraged to incorporate cardiovascular, strength, flexibility and balance activities into their routines, with flexibility regarding scheduling and combination of activity types. The design aims to maximise adherence while accounting for individual preferences and circumstances.

By embedding a rigorous process evaluation within the CANFit randomised feasibility trial, we aim to generate a rich understanding of how the intervention operates, how context influences outcomes and how exercise interventions might be effectively implemented into routine cancer care. This evaluation will integrate qualitative and quantitative findings to produce a nuanced understanding of intervention processes, supporting the interpretation of CANFit trial outcomes.

## Aims and objectives

### Aim

To explain how individual-level and team-level factors enabled or constrained fidelity of intervention delivery in the CANFit trial.

### Objectives

1. Assess whether the intervention was delivered as intended, unexpected consequences and the perceived relevance of the intervention to participants.
2. Analyse what was implemented and how: what was delivered, how much was delivered (number of sessions offered and received), efficiency of delivery and what constitutes usual care.
3. Develop an in-depth understanding of the acceptability of the intervention from the perspectives of participants and practitioners delivering the intervention and the key facilitators and constraints influencing acceptability
4. Assess the acceptability and appropriateness of trial processes from the perspectives of participants and participating sites
5. Understand how context affects implementation of the intervention.

## Methods and analysis

### Design

This is a hybrid type 1 study,^17^ where implementation research is embedded within a randomised controlled trial primarily focused on clinical outcomes. The hybrid design will enable the collection of explanatory implementation data alongside effectiveness outcomes, enhancing the interpretation of trial findings. A guiding framework will be used to focus the implementation evaluation and ensure consistent reporting of outcomes.

### Integration with main trial

Some implementation outcomes, particularly *acceptability*, will be partially assessed within the main trial protocol.^15^ At the 6-month follow-up (T1), participants will complete an 11-item survey based on the Theoretical Acceptability Framework, including Likert scale and open-ended questions. The process evaluation will build on and extend these findings by collecting additional data across multiple timepoints and from diverse stakeholder groups, including trainers, clinical staff and commissioners. Through methods such as semi-structured interviews, researcher field notes and trainer diaries, the process evaluation will generate in-depth insights into five implementation outcomes. These data are designed to complement the main trial outcomes and enhance understanding of how and why the intervention functions within its context.

### Guiding framework and outcomes

**Figure 1:**
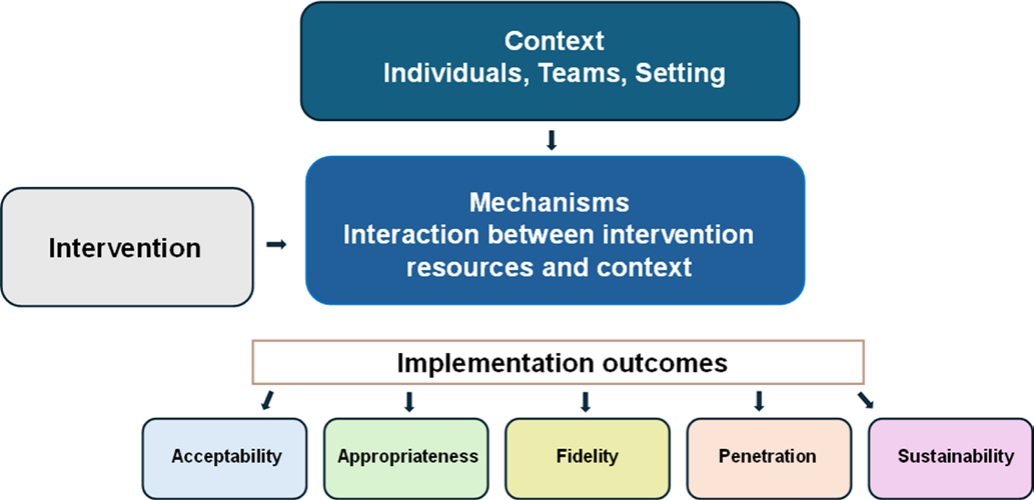
Conceptual framework guiding the CANFit process evaluation. Based on realist principles, the framework illustrates how context interacts with intervention resources to generate mechanisms that influence five implementation outcomes: acceptability, appropriateness, fidelity, penetration and sustainability, informed by realist evaluation principles and Proctor *et al*’s framework.^5 15^

A key consideration in process evaluation is defining successful implementation.^6^ Proctor *et al* describe eight core implementation outcomes that distinguish between intervention effectiveness and implementation effectiveness.^1^ Table 1 provides an integrated overview that maps implementation outcomes against stage of analysis, data collection methods, participant sources and study objectives. Outcomes were selected because they are most relevant to understanding feasibility, early-stage implementation and contextual fit in this trial. The conceptual framework guiding the process evaluation is summarised in figure 1.

As terminology across outcomes can overlap, clear definitions will be provided following Lengnick-Hall *et al*’s guidance to ensure consistency throughout the study.^2^ Flexibility has been built into data collection approaches to reflect the feasible nature of the trial.

The five outcomes will be the following:

- Acceptability.
- Appropriateness.
- Fidelity.
- Penetration.
- Sustainability.

#### Acceptability

Acceptability will be assessed to understand stakeholder attitudes towards the CANFit intervention, including perceptions of its burden, appeal and value.

#### Appropriateness

Appropriateness will be measured as the perceived fit, relevance, and compatibility of the CANFit intervention with participants’ needs and the clinical context. It will be assessed separately from acceptability to distinguish interventions that are acceptable but not necessarily appropriate or vice versa.

#### Fidelity

Fidelity will be evaluated through multiple components:

- Adherence: assessment of whether key intervention components will be delivered as intended.
- Dose exposure: data on the number of sessions offered and attended and overall intervention duration (first to last session).
- Quality of delivery: structured review of CANFit intervention delivery.
- Programme differentiation: capturing adaptations and modifications made during delivery.
- Participant responsiveness: participant engagement and feedback.

#### Penetration

Penetration will be assessed by calculating the proportion of eligible patients who participate in the intervention relative to the total eligible population and by assessing the proportion of trained providers who deliver the intervention, where feasible.

#### Sustainability

While sustainability is included as one of the five selected Proctor implementation outcomes, we note that the main study is in the feasibility phase; therefore, exploration of sustainability in the process evaluation will be limited to early feasibility considerations and perceptions, rather than a full assessment of long-term sustainability.

### Realist principles

The process evaluation will be informed by realist principles, which focus on understanding how, why, for whom and under what circumstances an intervention works.^18^ This approach will guide the design of data collection methods to capture the interplay between context and mechanisms that achieve (or fail to achieve) intended outcomes. Applying realist principles alongside the selected Proctor outcomes^1^ will enable the evaluation to generate explanatory insights into how contextual factors shape implementation success or failure, beyond simply measuring outcome metrics.

### Setting and participants

Since the process evaluation includes only those who enrolled in the trial or who declined or withdrew but consented to interview, the range of perspectives is dependent on the characteristics of this group. Within these constraints, purposive sampling will be used to capture diverse perspectives across a range of sociodemographic characteristics, including socioeconomic position, ethnicity, gender and participant’s ability to engage with digital or written materials (health literacy/digital literacy), to explore how these factors may influence acceptability and engagement.

The CANFit intervention will be delivered across multiple NHS sites in England, with seven sites currently open for recruitment and an overall target of at least ten sites. Recruitment began in February 2024 and will continue until the recruitment target is met. The clinical settings include oncology outpatient services and affiliated clinics, targeting adults diagnosed with lung, bowel or breast cancer who are considered at high risk of recurrence.

While participants will be recruited through NHS oncology outpatient services at participating sites, the CANFit intervention will be delivered primarily via remote online methods, including digital resources and virtual counselling sessions. This approach is designed to support flexible access across geographically diverse populations and accommodate patient preferences.

### Sample size

The process evaluation will use purposive sampling to capture diverse perspectives, including an estimated 48–60 patient interviews (approximately up to 10% of participants randomised to the trial), 8–12 interviews with people who decline to take part or withdraw, 15–20 staff interviews and 4–6 commissioner interviews. These initial ranges were determined pragmatically to ensure adequate representation of key stakeholder perspectives and variation across sites, while reflecting the relative size and anticipated accessibility of each group.

Quantitative process data (eg, session attendance and fidelity metrics) and trainer diaries will also be collected. Sample size will be guided by the goal of achieving data adequacy and thematic saturation, rather than statistical power calculations.^19^ Flexibility will be retained to adjust sample size if emergent findings indicate the need for additional or targeted interviews to ensure comprehensive insights.

Table 1 provides a summary of the participants’ groups, estimated sample sizes and recruitment timelines for the process evaluation.

**Table 1:** Summary of participant groups’ estimated sample sizes and recruitment timing for the process evaluation

Participant group	Description	Estimated sample size	Recruitment timing
Patients	Randomised trial participants	48–60 (~10% of trial sample)	During intervention and follow-up
Decliners/dropouts	Patients who decline or withdraw from the trial	8–12	At point of decline or withdrawal
Staff	Clinicians, trainers, research nurses involved in delivery	15–20	During trial recruitment and delivery
Commissioners	NHS commissioners overseeing cancer rehab services	4–6	Towards end of study, post-intervention

### Data collection

A mixed-methods approach will be used to collect process evaluation data across the five core implementation outcomes (table 2). Quantitative data will be obtained through participant questionnaires, trainer diaries, intervention website metrics, and screening and recruitment logs. Qualitative data will be gathered via semi-structured interviews, questionnaires, trainer diaries and researcher observations, exploring experiences of the intervention, acceptability, contextual factors and implementation processes.

**Table 2:** Mapping of implementation outcomes to stage of analysis, data collection methods, participant sources and study objectives

Implementation outcome	Stage of analysis	Data collection method	Who	Objectives
Acceptability	All stages	Surveys, interviews, trainer diaries, intervention website	Site stakeholders, participants receiving and trainers delivering intervention	1, 3, 4
Appropriateness	Early	Interviews, surveys, intervention website	Participants	4
Fidelity (adherence, dose exposure, quality of delivery, programme component differentiation and patient responsiveness)	All stages	Observation (field notes), trainer diaries and notes, interviews, intervention website feedback and surveys	Participants, site stakeholder and trainers	2, 5
Penetration	Mid-late stage	Recruitment logs and interviews	Individual providers	2
Sustainability	Late stage	Interviews	Individual providers	5

Data collection will occur throughout the study period and will draw on multiple sources and perspectives with attention to capturing diversity in participant experiences where feasible. Table 3 summarises the staff participant roles and their involvement in data collection activities. Ethnicity data will be drawn from the main trial dataset to support analysis of equity of reach. Other diversity-related insights (eg, digital access, language and cultural relevance) will be explored qualitatively through participant interviews. Below, we describe the specific data sources and collection approaches mapped to each of the five selected implementation outcomes.

**Table 3:** Summary of key study roles and responsibilities in recruitment and intervention delivery

Role	Description
Research nurse	Identify eligible patients at multidisciplinary team (MDT) meetings
Trainers	Contact and gain patient consent; provide exercise support and counselling
Site principal investigator (PI)	Act as a champion to support all clinicians on site
Commissioner	Decide if intervention is feasible in daily practice
Clinician	Hold initial discussion with eligible patient; offer study leaflet (acceptance of leaflet gives consent to be contacted for recruitment)

#### Acceptability

Data will be collected at different timepoints to capture both anticipated acceptability (early stages) and experienced acceptability (mid-to-late stages). Data sources will include participant and trainer questionnaires, semi-structured interviews, trainer diaries and feedback gathered through the intervention website.

#### Appropriateness

Appropriateness will be assessed using participant questionnaires, semi-structured interviews, and trainer diaries, focusing on the perceived fit and relevance of the intervention.

##### Fidelity

Fidelity will be evaluated through several components:

- Adherence: Assessment of whether key intervention components are delivered as intended, using observation checklists and trainer self-assessment tools.
- Quality of delivery: Review of recorded counselling sessions (where feasible) and trainer reflections captured in diaries.
- Participant responsiveness: Insights gathered through participant questionnaires and interviews. Exploring factors that influenced engagement and relevance, including where possible, participants' confidence engaging with digital or written materials (as a proxy for health and/or digital literacy).

Trainer diaries will provide contemporaneous data, with brief weekly entries submitted via the Qualtrics platform, combining open-ended and closed-ended responses.

### Penetration

Penetration will be assessed using screening and recruitment logs to track the proportion of eligible patients participating in the intervention. The proportion of trained providers delivering the intervention will also be monitored through site-level tracking.

### Sustainability

As formal assessment of long-term sustainability beyond the scope of this process evaluation is not possible, exploratory data on sustainability perceptions will be collected through qualitative interviews with clinicians, commissioners and trainers during the latter stages of this process evaluation.

### Data analysis

#### Data management

Interviews will be audio-recorded using a digital device, and online interviews will also be video-recorded. Recordings will be transcribed either via the Microsoft Teams automatic transcription service, authorised process evaluation researchers, or an approved external transcription company contracted by the university (under confidentiality agreements). Transcripts will be anonymised to remove any potentially identifying information, after which recordings will be deleted. Files will be securely transferred using encrypted methods (via Box) and stored at the University of Hull, accessible only to designated members of the study team.

#### Quantitative analysis

Quantitative process data (eg, session attendance, adherence rates, fidelity metrics and questionnaire responses) will be summarised using descriptive statistics. These will include frequencies, proportions, means and SD, as appropriate. Analyses will focus on describing implementation patterns across sites and participant groups rather than formal hypothesis testing.

#### Qualitative analysis

Qualitative data analysis will occur concurrently with data collection, allowing emerging insights or theoretical concepts to inform subsequent interviews. Analysis will be primarily conducted by one researcher and reviewed by a second to ensure that gaps and conflicting evidence are identified and explored during ongoing data collection.

A combined deductive and inductive framework analysis approach will be employed,^20^ focusing on the following:

- How the intervention will be received (acceptability and relevance).
- How the intervention mechanisms will operate.
- How context will influence intervention implementation and outcomes.

#### Data integration

**Figure 2:**
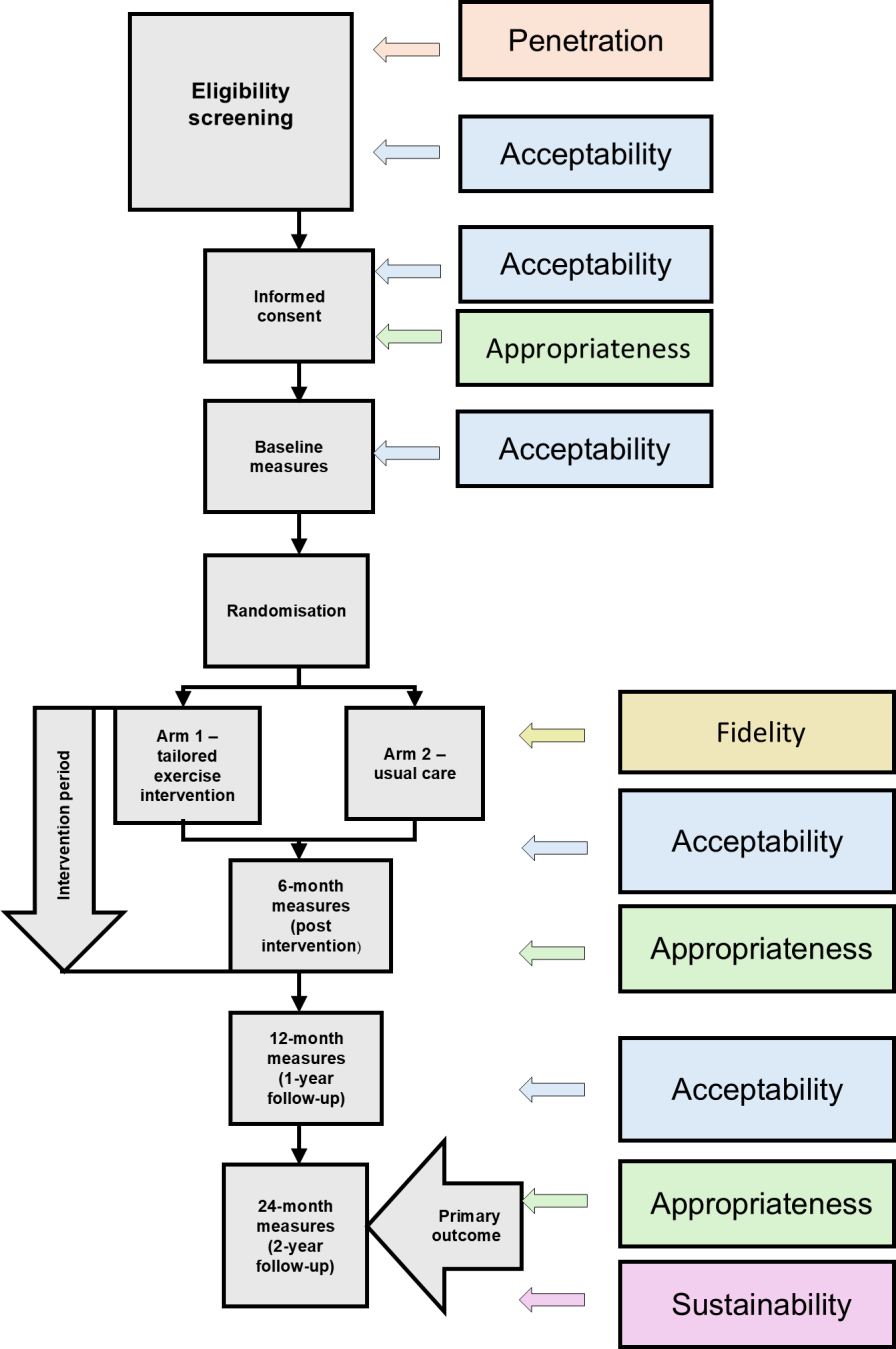
Mapping of process evaluation outcomes onto the main trial flow. This figure illustrates how five core implementation outcomes—acceptability, appropriateness, fidelity, penetration and sustainability—are assessed at defined stages of the CANFit trial. Each coloured box corresponds to a specific outcome measured through pre-specified methods as described in the protocol. Mapping outcomes across the trial flow supports systematic integration of the process evaluation and enables exploration of how implementation factors interact with trial delivery.

A convergent mixed-methods approach will be adopted, continuously triangulating qualitative and quantitative datasets. Data will be mapped into matrices to empirically refine a conceptual framework, using a Context-Mechanism-Outcome (CMO) configuration (figure 2). This will help understand how the implementation context shapes delivery mechanisms and their intended and unintended outcomes across cancer types.

At study completion, we aim to produce a refined understanding of the intervention’s function across populations and an updated logic model. Findings from the process evaluation will be presented to the trial management group and steering committee following analysis. Emergent process findings relating to trial implementation (eg, issues with recruitment, adherence and fidelity) will also be fed back in real-time during the trial to inform proactive adaptations if necessary.

### Patient and public involvement

The CANFit trial incorporated patient and public involvement (PPI) from the inception of the grant application. A patient advisory group has been established, with PPI members contributing to both the trial management group and the study steering committee. PPI has informed the development of the intervention, study materials and dissemination strategies. Ongoing consultation with PPI representatives will continue throughout the conduct and analysis of both the main trial and the process evaluation. Full details of PPI activities related to the main trial are reported separately in the main trial protocol^15^

### Reflexivity

The process evaluation will be conducted independently from the main trial to maintain objectivity. It will be led by an implementation scientist and a mixed-methods researcher, whose multidisciplinary backgrounds in nursing and psychology will support the exploration of diverse perspectives. A dedicated research fellow, with prior research experience in cancer and process evaluations, will maintain contemporaneous field notes, a reflexive diary and a log of emerging ideas and theories throughout data collection and analysis to enhance the rigour and transparency of the evaluation.^21 22^

## Discussion

This process evaluation will provide insights into the implementation and contextual factors influencing delivery and uptake of the CANFit intervention. It complements the main basket-design randomised controlled feasibility trial evaluating a personalised, remote exercise programme for people diagnosed with cancer at increased risk of recurrence. The basket design, relatively novel in exercise oncology, allows examination of both shared and cancer type-specific challenges. Combined with the realist-informed process evaluation, the study aims to determine which components are feasible, for whom and under what conditions to inform potential adaptation and the design of a future definitive trial.

Exercise interventions for people diagnosed with cancer are complex and context-dependent, particularly across tumour types and healthcare settings. Existing evidence is largely observational or limited to lower-risk populations, with few trials integrating clinical and implementation science approaches. This hybrid type one design addresses this gap by embedding the study of implementation processes alongside clinical outcomes, enabling richer interpretation to guide next-stage research.

The focus on five implementation outcomes—acceptability, appropriateness, fidelity, penetration and sustainability—allows assessment of stakeholder experiences, delivery alignment and uptake.^23^ A convergent mixed-methods design supports systematic triangulation,^24 25^ and Framework Analysis ensures flexibility with consistency in coding.^20^ Findings will inform the planning of future trials and strengthen the evidence base for exercise interventions in people diagnosed with cancer.^26^,^28^

## Ethics and dissemination

This study gained ethical approvals from Hull York Medical School (ID: 23/SS/0060) and the UK NHS Health Research Authority (ID: 327663) and adheres to the Declaration of Helsinki. Study findings will be submitted for publication in high-impact cancer-related or exercise science journals, presentation at relevant national and international conferences, press releases where appropriate and dissemination activities that will be discussed and decided on with the patient advisory group to ensure our results are translatable to patient and public groups.

Results will also be shared with participating sites and, where appropriate, with study participants through lay summaries.

Dissemination activities will aim to support the wider adoption of effective exercise interventions in oncology settings and contribute to the broader implementation science evidence base for care of people diagnosed with cancer.

This protocol has been developed and reported in accordance with the SPIRIT (Standard Protocol Items: Recommendations for Interventional Trials) 2013 statement.
