# Supplementary material for: How can a remotely delivered, personalised physical activity intervention for people with high risk of breast, lung and bowel cancer recurrence be implemented in England? Protocol for a mixed-methods process evaluation embedded in a feasibility basket trial
Source: BMJ Open. 2026 May 24;16(5):e112103. doi: 10.1136/bmjopen-2025-112103 (PMC13202128; doi:10.1136/bmjopen-2025-112103)
Supplement: Supplementary file 1 [file bmjopen-2025-112103.reviewer_comments.pdf]

PEER REVIEW HISTORY

BMJ Open publishes all reviews undertaken for accepted manuscripts. Reviewers are asked to complete a checklist review form and are provided with free text boxes to elaborate on their assessment. These free text comments are reproduced below.

ARTICLE DETAILS

Title (Provisional)

How can a remotely-delivered, personalised physical activity intervention for people with high risk of breast, lung, and bowel cancer recurrence be implemented in England? Protocol for a mixed-methods process evaluation embedded in a feasibility basket trial

Authors

Jackson, Gillian; Ferdowsi, Lubna; Twiddy, Maureen; Pearson, Mark; Bullock, Alex F; Cohen, Judith; Huang, Chao; Lind, Michael; Saxton, John; Wilson, Caroline; Forbes, Cynthia

---

VERSION 1 - REVIEW

**Reviewer**	**1**
**Name**	**Ibbotson, Tracy**
**Affiliation**	**University of Glasgow, Institute of Health and Wellbeing**
**Date**	**02-Jan-2026**
**COI**	**None**

The CANFit intervention addresses an important unmet need for people living with and beyond cancer who are at increased risk of recurrence. The intervention is well conceived, with a strong emphasis on individual tailoring and a tapered support model that is likely to encourage sustained engagement in physical activity. The inclusion of a process evaluation is a particular strength and offers valuable opportunities to understand implementation, engagement, and contextual influences on outcomes.

The protocol describes robust data collection, including a range of socioeconomic variables, which will support exploration of reach and engagement. The process evaluation could be further strengthened by more explicitly outlining how the acceptability of the physical activity intervention will be examined across different sociodemographic groups. Greater clarity on how experiences may vary by socioeconomic position, literacy, or ethnicity would enhance the contribution of the evaluation.

The use of primarily remote, online delivery methods—including digital resources and virtual counselling—supports flexible access across geographically diverse populations and aligns

well with contemporary models of care. To maximise inclusivity, the authors may wish to provide additional detail on how potential barriers related to digital access, health or digital literacy, language, and cultural appropriateness will be considered, particularly for participants from diverse ethnic backgrounds.

Finally, while recruitment processes are clearly described, the protocol would benefit from further discussion of how recruitment and data collection will enable assessment of equity of reach. In particular, the absence of ethnicity data limits the ability to fully explore inclusivity and differential engagement. Addressing this, or providing a rationale and reflecting on the implications, would strengthen the interpretation and generalisability of the findings.

Overall, this is a strong and timely protocol, and with minor enhancements around inclusivity and equity, the process evaluation has the potential to make an important contribution to the field.

**Reviewer**	**2**
**Name**	**Dack, Kyle**
**Affiliation**	**University of Bristol, MRC Integrative Epidemiology Unit**
**Date**	**23-Jan-2026**
**COI**	**None**

Statistical review summary:

This protocol describes an implementation study which is to be embedded in a larger basket RCT. I cannot comment much on the statistical analyses as they were not described in the main text. It appears they will be descriptive and supplementary in nature, so there is a low risk of concern, but in this protocol the quantitative element does appear underspecified. Other than this, the design and clarity of the methods appears clear and I have only minor comments.

Abstract:

The abstract is clear and reflects the protocol well. However, along with a sentence in the strengths/limitations “The hybrid type 1 design enables exploration of implementation outcomes alongside clinical effectiveness”, it may give readers the impression that clinical effectiveness is part of this study. I suggest adding an explanation similar to the first sentence in the methods, to make a clearer distinction between the two elements of the RCT.

Introduction:

Very clear and no comments.

Methods

Figure 1: This figure is lower resolution than Figure 2, and the smallest text (in the bottom row) is quite blurry to read.

Sustainability subheading: It is remarked in this section that this outcome will be explored to only a limited extent within the feasibility phase. Does this mean there are further process evaluation studies planned? Generally it is not clear to me whether "feasibility" in this manuscript is referring to the host RCT, this embedded study, or both. A general overview in the introduction of future planned studies may help to clarify this.

Sample sizes: There are different target numbers for different subgroups of the study, as shown in table 2. Not using formal sample size estimation is reasonable, but how were the sizes determined. E.g. why 4-6 commissioners and 8-12 decliners, and not vice versa? Are the numbers intended to represent approximately the same proportion of the overall study participants / staff? A brief sentence to explain the rationale behind the numbers may be helpful.

There is description of the qualitative analysis and mixed methods approach, but not details of any quantitative approach. The only mention of quantitative analysis is in the abstract – "Quantitative data will be analysed descriptively", but not in the main methods text. I suggest a brief section similar to the qualitative section to explain which measures will be summarized and how.

VERSION 1 - AUTHOR RESPONSE

**Reviewer 1**		
The CANFit intervention addresses an important unmet need for people living with and beyond cancer who are at increased risk of recurrence. The intervention is well conceived, with a strong emphasis on individual tailoring and a tapered support model that is likely to encourage sustained engagement in physical activity. The inclusion of a process evaluation is a particular strength and offers valuable opportunities to understand implementation, engagement, and	We thank the reviewer for their supportive comment.	N/A

contextual influences on outcomes.		
The protocol describes robust data collection, including a range of socioeconomic variables, which will support exploration of reach and engagement. The process evaluation could be further strengthened by more explicitly outlining how the acceptability of the physical activity intervention will be examined across different sociodemographic groups. Greater clarity on how experiences may vary by socioeconomic position, literacy, or ethnicity would enhance the contribution of the evaluation.	We have revised the settings and participants section to clarify that the process evaluation sample includes trial participants as well as individuals who declined or withdrew but consented to interview. As such, the range of perspectives is dependent on this group. Within these constraints, purposive sampling will aim to capture diversity across characteristics such as socioeconomic position, ethnicity, gender and participant's ability to engage with digital and written materials (as a proxy for health/digital literacy), where feasible. These considerations are also reflected in the participant responsiveness section. We have not repeated this detail in other sections to avoid unnecessary duplication.	Pages 9, 10,11
The use of primarily remote, online delivery methods—including digital resources and virtual counselling—supports flexible access across geographically diverse populations and aligns well with contemporary models of care. To maximise inclusivity, the authors may wish to provide additional detail on how potential barriers related to digital access, health or digital	Ethnicity data will be collected at baseline within the main feasibility trial and will be available to the process evaluation for use in assessing equity of reach. Other aspects of diversity (e.g., digital access, health/digital literacy and cultural relevance) will be explored through purposive sampling and interview data. We have updated the Data Collection and Limitations sections of the manuscript to	Pages 3, 10

literacy, language, and cultural appropriateness will be considered, particularly for participants from diverse ethnic backgrounds.	reflect this and to clarify both the scope and constraints of our equity focused analysis.	
Finally, while recruitment processes are clearly described, the protocol would benefit from further discussion of how recruitment and data collection will enable assessment of equity of reach. In particular, the absence of ethnicity data limits the ability to fully explore inclusivity and differential engagement. Addressing this, or providing a rationale and reflecting on the implications, would strengthen the interpretation and generalisability of the findings.	Thank you. As outlined in the previous responses, ethnicity data are collected within the main feasibility trial and will be used within the process evaluation to assess equity of reach. Recruitment and data collection therefore enable examination of reach using trial demographic data, alongside qualitative exploration of inclusivity and differential engagement across participant groups. This has been clarified at the relevant points in the revised manuscript, as noted previously.	Pages 3,9,10,11
Overall, this is a strong and timely protocol, and with minor enhancements around inclusivity and equity, the process evaluation has the potential to make an important contribution to the field.	We thank the reviewer for their positive feedback and are pleased that the protocol is recognised as a strong and timely contribution to the field.	N/A
**Reviewer 2**		
This protocol describes an implementation study which is to be embedded in a larger basket RCT. I cannot comment much on the statistical analyses as they were not described in the main text. It appears they will be descriptive and	Thank you for the positive feedback on the clarity of the design and methods. In response to the comment regarding the quantitative component, we have added a brief quantitative analysis subsection to the Methods to clarify the types of process	Page 12

supplementary in nature, so there is a low risk of concern, but in this protocol the quantitative element does appear underspecified. Other than this, the design and clarity of the methods appear clear, and I have only minor comments	measures that will be summarised and the descriptive approach to analysis.	
Abstract:<br>The abstract is clear and reflects the protocol well. However, along with a sentence in the strengths/limitations "The hybrid type 1 design enables exploration of implementation outcomes alongside clinical effectiveness", it may give readers the impression that clinical effectiveness is part of this study. I suggest adding an explanation similar to the first sentence in the methods, to make a clearer distinction between the two elements of the RCT.	We agree that the previous wording in the strengths and limitations section could have implied that clinical effectiveness was evaluated within this study. This statement has been revised to remove reference to clinical effectiveness and avoid potential misinterpretation.	Page 3
Introduction:<br>Very clear and no comments.		N/A
Methods<br>Figure 1: This figure is lower resolution than Figure 2, and the smallest text (in the bottom row) is quite blurry to read.	The figure has been revised to improve clarity and resolution	Page 20
Sustainability subheading:<br>It is remarked in this section that this outcome will be explored to only a limited extent within the feasibility phase. Does this mean there are further process	We have clarified that 'feasibility' refers to the host CANFit randomised feasibility trial and have revised the manuscript to ensure this is stated explicitly at first mention and within	Pages 4, 5,9,12

evaluation studies planned? Generally, it is not clear to me whether "feasibility" in this manuscript is referring to the host RCT, this embedded study, or both. A general overview in the introduction of future planned studies may help to clarify this.	the Background section. The 2 sustainability subsections have also been amended to clarify that formal long-term sustainability is beyond the scope of this process evaluation.	
Sample sizes: There are different target numbers for different subgroups of the study, as shown in table 2. Not using formal sample size estimation is reasonable, but how were the sizes determined. E.g. why 4-6 commissioners and 8-12 decliners, and not vice versa? Are the numbers intended to represent approximately the same proportion of the overall study participants / staff? A brief sentence to explain the rationale behind the numbers may be helpful.	Thank you for raising this point. In response, we have added a brief explanation to the sample size section to clarify the rationale for the subgroup target ranges. These were determined pragmatically to ensure adequate representation of key stakeholder perspectives and variation across sites, while reflecting the relative size and anticipated accessibility of each group.	Page 10<br>In sample size
There is description of the qualitative analysis and mixed methods approach, but not details of any quantitative approach. The only mention of quantitative analysis is in the abstract – "Quantitative data will be analysed descriptively", but not in the main methods text. I suggest a brief section similar to the qualitative section to explain which measures will be summarized and how.	A brief quantitative analysis subsection has now been added to the Methods to clarify the types of quantitative process measures that will be summarised and the descriptive approach to analysis.	Page 12
